# Low serum magnesium is associated with poor functional outcome in acute ischemic stroke or transient ischemic attack patients

**DOI:** 10.1111/cns.14020

**Published:** 2022-11-22

**Authors:** Qin Xu, Lele Hu, Lu Chen, Hao Li, Xue Tian, Yingting Zuo, Yijun Zhang, Xiaoli Zhang, Ping Sun, Yongjun Wang, Xia Meng, Anxin Wang

**Affiliations:** ^1^ Department of Neurology, Beijing Tiantan Hospital Capital Medical University Beijing China; ^2^ China National Clinical Research Center for Neurological Diseases Beijing Tiantan Hospital, Capital Medical University Beijing China; ^3^ The Second People's Hospital of Guiyang Guizhou China; ^4^ Department of Neurology ZiBo Central Hospital Zibo China; ^5^ Department of Epidemiology and Health Statistics School of Public Health, Capital Medical University Beijing China; ^6^ Beijing Municipal Key Laboratory of Clinical Epidemiology Beijing China; ^7^ Advanced Innovation Center for Human Brain Protection Capital Medical University Beijing China; ^8^ Center for Excellence in Brain Science and Intelligence Technology Chinese Academy of Sciences Shanghai China

**Keywords:** mortality, poor functional outcome, serum magnesium, stroke

## Abstract

**Aim:**

The association between magnesium and outcomes after stroke is uncertain. We aimed to investigate the association of serum magnesium with all‐cause mortality and poor functional outcome.

**Methods:**

We included patients with acute ischemic stroke (AIS) or transient ischemic attack (TIA) from the China National Stroke Registry III. We used Cox proportional hazards model for all‐cause mortality and logistic regression model for poor functional outcome (modified Rankin Scale [mRS] 2–6/3–6) to examine the relationships.

**Results:**

Among the 6483 patients, the median (interquartile range) magnesium was 0.87 (0.80–0.93) mmol/L. Patients in the first quartile had a higher risk of mRS score 3–6/2–6 at 3 months (adjusted odds ratio [OR]: 1.30; 95% confidence interval [CI]: 1.02, 1.64; adjusted OR: 1.29; 95% CI: 1.04–1.59) compared with those in the fourth quartile. Similar results were found for mRS score 26 at 1 year. The age‐ and sex‐adjusted hazard ratio (HR) with 95% CI in first quartile magnesium was 1.40 (1.02–1.93) for all‐cause mortality within 1 year, but became insignificant (HR: 1.03; 95% CI: 0.71–1.50) after adjusting for potential variables.

**Conclusions:**

Low serum magnesium was associated with a high risk of poor functional outcome in patients with AIS or TIA.

## Graphical abstract

Low serum magnesium was associated with a high risk of poor functional outcome at 3 months and 1 year in patients with ischemic stroke or transient ischemic attack. Measurement of serum magnesium at admission might be helpful for identifying patients at high risk of poor outcomes.

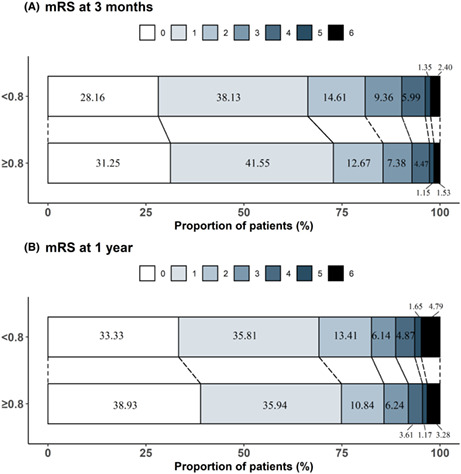

## 1 INTRODUCTION

Magnesium, the second most abundant intracellular electrolyte after potassium, is an essential mineral for human health.^1^, ^2^ Magnesium comes from many foods, such as green leafy vegetables, fruits, dairy products, and nuts.^3^ Despite the abundant distribution of magnesium in foods, however, magnesium deficiency is common worldwide, even in developed countries.^4^ According to the 2015–2020 dietary guidelines for Americans, most Americans consume less than the average daily requirement of magnesium.^5^

Magnesium plays an important role in the cardiovascular system through its antithrombosis and antiinflammatory effects, as well as the regulation of blood pressure, endothelial cell function, glucose, and insulin metabolism.^6^, ^7^ Previous observational studies have shown that the serum magnesium was inversely associated with the risk of a wide range of diseases, including cardiovascular disease (CVD),^3^, ^8^, ^9^ type 2 diabetes mellitus,^10^, ^11^ metabolic syndrome,^12^ arterial hypertension,^13^, ^14^ and chronic kidney disease,^13^, ^15^ and also associated with coronary heart disease mortality.^16^ Based on the pathogenesis of magnesium and CVD, studies found that serum magnesium was associated with stroke risk.^17^, ^18^ Experimental animal studies show that magnesium has neuroprotective properties.^19^, ^20^ Low magnesium at the onset of stroke may accelerate penumbral compromise, resulting in early neurologic deterioration and more severe stroke outcome.^21^ Some epidemiological studies have addressed the relationship between serum magnesium and prognosis in patients with acute ischemic stroke (AIS), but reported conflicting results.^22^, ^23^, ^24^, ^25^ You et al. found that low magnesium levels were independently associated with in‐hospital mortality in AIS patients.^22^ But, others found a null association.^25^ Moreover, the impact of serum magnesium concentration on functional outcome and long‐term mortality after stroke is not yet sufficiently elucidated.

Thus, we aimed to evaluate the association between serum magnesium levels and clinical outcomes after AIS or transient ischemic attack (TIA), including all‐cause mortality and poor functional outcome at 3 months and 1 year.

## 2 METHODS

### 2.1 Study design and population

We derived data from the Third China National Stroke Registry (CNSR‐III), which is a large‐scale nationwide, multicenter, prospective registry. Details of the design and procedure of the CNSR‐III have been previously described.^26^ Briefly, the CNSR‐III enrolled patients with acute ischemic cerebrovascular events who presented to hospitals if they met the following criteria: aged 18 years and older; diagnosis of ischemic stroke or TIA within 7 days from the onset of symptoms to enrollment; informed consent from the patient or legally authorized representatives. A total of 15,166 patients were recruited consecutively between August 2015 and March 2018 from 201 hospitals in 22 provinces and 4 municipalities in China. The study was conducted following the guidelines laid down in the Declaration of Helsinki, and all procedures involving humans were approved by the ethics committee of Beijing Tiantan Hospital and participant hospitals. Written informed consent was obtained from the patients or their legally authorized representatives.

### 2.2 Data collection

Trained research coordinators at each center collected baseline information via a face‐to‐face interview or medical records, including age, sex, body mass index (BMI; calculated as the weight in kilograms divided by the square of the height in meters, kg/m^2^), smoking and alcohol consumption status, medical history, prestroke modified Rankin Score (mRS), the etiological classification conducted by the Trial of Org 10172 in Acute Stroke Treatment (TOAST) criteria,^27^ the National Institutes of Health Stroke Scale (NIHSS) score at admission and at discharge, and medications in hospital.

### 2.3 Sample collection and measurement of serum magnesium

Fasting blood samples were collected within 24 h of admission. Magnesium was measured in serum by a colorimetric method using unfrozen samples at each participating hospital. All measurements were performed by laboratory personnel blinded to patients' clinical situations. The blood samples were frozen in a cryotube at −80°C in refrigerator, then were transported through cold chain to the central laboratory in Beijing Tiantan Hospital. Total cholesterol (TC), high‐density lipoprotein (HDL), low‐density lipoprotein (LDL), triglyceride (TG), and serum creatinine were tested centrally. The estimated glomerular filtration rate (eGFR) was calculated by the CKD‐EPI China equation with a coefficient of 1.1 for the Chinese population.^28^ eGFR_CKD EPI(CN)_ = 141 × min(SCr/k,1)^α^ × max (SCr/k,1)^−1.209^ × 0.993^Age^ × 1.018 (if female) × 1.1, where SCr is serum creatinine, k is 0.7 for females and 0.9 for males, α is −0.329 for females and −0.411 for males, min is the minimum of SCr/k or 1, and max indicates the maximum of SCr/k or 1.

### 2.4 Outcome assessment

The clinical outcomes were obtained by trained research coordinators who were blinded to subjects' baseline characteristics, through a face‐to‐face interview at 3 months and through telephone at 1 year after symptom onset. Adverse outcomes in our study included all‐cause mortality and poor functional outcome at 3 months and 1‐year follow‐up. All‐cause mortality was either confirmed by a death certification from the attended hospital or the local citizen registry. Modified Rankin Scale (mRS) score ranged from 0 (no symptoms) to 6 (death), and poor functional outcome was defined as mRS score of 3–6 or 2–6.

### 2.5 Statistical analysis

We divided patients into four groups according to quartiles of serum magnesium and two groups by the cut‐off value of 0.8 according to the previous study.^16^ We used Kolmogorov‐Smirnov test to assess the distribution of continuous variables, then they were expressed as medians with interquartile ranges (IQRs) because of nonnormality and tested using the Kruskal‐Wallis test for their differences across the four groups by quartiles of serum magnesium. Categorical variables were described as frequencies (percentages) and tested using the Chi‐square test or Fisher's exact test.

The association of serum magnesium groups with all‐cause mortality and poor functional outcome at 3 months and 1 year was assessed. For all‐cause mortality, we used the Kaplan–Meier method to estimate the cumulative hazards, and the difference across two groups of serum magnesium was calculated by the log‐rank test. Hazard ratios (HRs) and 95% confidence intervals (CIs) were estimated by Cox proportional hazards models. The proportionality assumption was assessed by adding a time‐dependent covariate with the interaction of magnesium and a logarithmic function of survival time in the Cox Model. For poor functional outcome, odds ratios (ORs) with 95% CIs were estimated by logistic regression models. The robust sandwich estimates of the variance‐covariance matrix were used to account for the clustering of patients within each of the hospitals. We fitted three adjusted models. Model 1 was adjusted for age and sex. Model 2 was further adjusted for body mass index, current smoker, current alcohol drinking, disease history (diabetes, atrial fibrillation, and coronary heart disease), the NIHSS score at admission, stroke etiology, anticoagulant agents, cholesterol‐lowering agents, hypoglycemic agents, and pulmonary infection. Model 3 was additionally adjusted for laboratory tests, including TC, TG, HDL, LDL, eGFR, fasting plasma glucose (FPG), and hospital stay. We performed a test for linear trend by treating the median magnesium value of each quartile as a continuous variable in each model. We further performed two sensitivity analyses. First, we additionally adjusted for serum sodium, potassium, and calcium levels to test whether the association of magnesium with any of the outcomes could be explained by a concomitant electrolyte disorder. We also excluded patients with diabetes mellitus and abnormal kidney function (defined as eGFR ≤ 60 ml/min per 1.73 m^2^) in order to explore potential effect modification by diabetes mellitus and chronic kidney disease. Furthermore, to capture the dose‐response relationship, we used restricted cubic splines with four knots at the 5th, 35th, 65th, and 95th percentiles of magnesium after adjusting all potential variables. Subgroup analysis was performed for associations between two groups of magnesium and outcomes according to age, sex, BMI, current smoker, current alcohol drinking, and stroke severity accessed by NIHSS score at admission and at discharge with an interaction test.

All analyses were performed using SAS software version 9.4 (SAS Institute Inc) at a two‐tailed α of 0.05.

## 3 RESULTS

### 3.1 Baseline characteristics

Of 15,166 patients, 8471 participants without serum magnesium and 212 patients without available mRS score at 3 months or 1 year were excluded. Thus, a total of 6483 patients were included in the final analysis. The baseline characteristics between included and excluded patients were approximately balanced, except that the included patients were more likely to be older, current nondrinkers, had a higher prevalence of hypertension, atrial fibrillation and pulmonary infection, a lower prevalence of stroke or TIA, dyslipidemia, and peripheral vascular disease, a shorter hospital stay, a lower TC and eGFR, a higher LDL, FPG, and NIHSS score, received more antihypertensive agents, anticoagulant agents, rt‐PA intravenous thrombolytic, and less cholesterol‐lowering agents (Table S1).

Among the 6483 patients, the median age was 63.0 years (IQR: 55.0–71.0), 32.02% were women, and 34.98% were current alcohol drinkers. Table 1 shows the baseline characteristics of the patients by four groups of serum magnesium concentration. The median serum magnesium level was 0.87 (IQR: 0.80–0.93) mmol/L. Patients in the lowest quartile magnesium group had more current alcohol drinkers, a higher prevalence of medical history, such as diabetes and atrial fibrillation, a higher NIHSS score at admission, a higher proportion of ischemic stroke and pulmonary infection, a higher FPG concentration, but a lower TC, HDL, and LDL concentration, and received more anticoagulant agents and hypoglycemic agents.

**TABLE 1:** Baseline characteristics according to quartiles of serum magnesium

Characteristics	Total	Quartiles of serum magnesium				*p* value
		Quartile1 (<0.80 mmol/L)	Quartile2 (0.80–0.87 mmol/L)	Quartile3 (0.87–0.93 mmol/L)	Quartile4 (≥0.93 mmol/L)	
No. of the patients	6483	1335	1848	1591	1709	
Serum magnesium, median (IQR), mmol/L	0.87 (0.80–0.93)	0.75 (0.71–0.78)	0.83 (0.81–0.85)	0.89 (0.88–0.91)	0.98 (0.95–1.03)	
Age, median (IQR), years	63 (55–71)	63 (54–71)	63 (55–71)	63 (56–70)	64 (55–71)	0.571
Women, *n* (%)	2076 (32.02)	410 (30.71)	606 (32.79)	483 (30.36)	577 (33.76)	0.114
BMI, median (IQR), kg/m^2^	24.49 (22.49–26.57)	24.22 (22.19–26.44)	24.49 (22.50–26.57)	24.49 (22.60–26.50)	24.49 (22.53–26.67)	0.120
Current smoker, *n* (%)	2005 (30.93)	439 (32.88)	561 (30.36)	504 (31.68)	501 (29.32)	0.160
Current alcohol drinking, *n* (%)	2842 (43.84)	619 (46.37)	807 (43.67)	712 (44.75)	704 (41.19)	0.031*
Medical history, *n* (%)						
Hypertension	4128 (63.67)	860 (64.42)	1171 (63.37)	1014 (63.73)	1083 (63.37)	0.926
Stroke or TIA	1501 (23.15)	304 (22.77)	435 (23.54)	382 (24.01)	380 (22.24)	0.630
Diabetes	1516 (23.38)	435 (32.58)	444 (24.03)	328 (20.62)	309 (18.08)	<0.001*
Dyslipidemia	444 (6.85)	88 (6.59)	125 (6.76)	100 (6.29)	131 (7.67)	0.434
Atrial fibrillation	504 (7.77)	138 (10.34)	148 (8.01)	115 (7.23)	103 (6.03)	<0.001*
Coronary heart disease	681 (10.50)	149 (11.16)	212 (11.47)	150 (9.43)	170 (9.95)	0.174
Heart failure	45 (0.69)	10 (0.75)	14 (0.76)	12 (0.75)	9 (0.53)	0.815
Peripheral vascular disease	38 (0.59)	5 (0.37)	16 (0.87)	8 (0.50)	9 (0.53)	0.284
Renal insufficiency	50 (0.77)	13 (0.97)	13 (0.70)	12 (0.75)	12 (0.70)	0.816
Admission stroke data						
Time from onset to hospital admission, h	11 (3–31)	11 (3–34)	11 (3–30)	12 (3–33)	11 (3–31)	0.864
hospital stay, days	13 (10–15)	13 (10–16)	13 (10–16)	13 (10–15)	13 (10–15)	0.051
NIHSS score at admission, median (IQR)	3 (1–6)	3 (2–6)	3 (1–6)	3 (1–6)	3 (1–6)	0.008*
Prestroke mRS score 2–5, *n* (%)	550 (8.48)	116 (8.69)	169 (9.15)	124 (7.79)	141 (8.25)	0.530
Stroke subtype, *n* (%)						
Ischemic stroke	6044 (93.23)	1280 (95.88)	1731 (93.67)	1468 (92.27)	1565 (91.57)	<0.001*
TIA	439 (6.77)	55 (4.12)	117 (6.33)	123 (7.73)	144 (8.43)	
Stroke etiology, *n* (%)						
Large‐artery atherosclerosis	1570 (24.22)	315 (23.60)	470 (25.43)	367 (23.07)	418 (24.46)	0.348
Cardioembolism	450 (6.94)	111 (8.31)	128 (6.93)	102 (6.41)	109 (6.38)	
Small‐vessel occlusion	1259 (19.42)	246 (18.43)	352 (19.05)	332 (20.87)	329 (19.25)	
Other determined etiology	105 (1.62)	20 (1.50)	36 (1.95)	20 (1.26)	29 (1.70)	
Undetermined etiology	3099 (47.80)	643 (48.16)	862 (46.65)	770 (48.40)	824 (48.22)	
Laboratory data, median (IQR)						
TC, mmol/L	4.24 (3.52–5.05)	4.19 (3.45–5.00)	4.24 (3.49–5.04)	4.20 (3.53–4.99)	4.31 (3.61–5.15)	0.011*
TG, mmol/L	1.37 (1.02–1.93)	1.38 (1.01–1.99)	1.38 (1.02–1.92)	1.33 (1.02–1.84)	1.41 (1.04–1.97)	0.083
HDL, mmol/L	1.09 (0.91–1.30)	1.05 (0.88–1.27)	1.08 (0.91–1.29)	1.10 (0.92–1.31)	1.12 (0.94–1.33)	<0.001*
LDL, mmol/L	2.52 (1.91–3.22)	2.43 (1.83–3.15)	2.53 (1.91–3.23)	2.50 (1.92–3.17)	2.60 (1.96–3.31)	0.003*
eGFR, ml/min/1.73 m^2^	94.9 (82.0–104.2)	95.3 (80.8–104.9)	95.5 (83.0–104.9)	94.9 (82.5–103.9)	94.1 (81.3–102.9)	0.088
FPG, mmol/L	5.6 (4.94–7)	5.87 (5.02–8.39)	5.63 (4.97–7.14)	5.54 (4.92–6.76)	5.47 (4.9–6.44)	<0.001*
Medications in hospital, *n* (%)						
Antihypertensive agents	3143 (48.66)	674 (50.75)	898 (48.83)	772 (48.58)	799 (46.92)	0.219
Antiplatelet agents	6273 (97.12)	1285 (96.76)	1784 (97.01)	1542 (97.04)	1662 (97.59)	0.555
Anticoagulant agents	761 (11.78)	184 (13.86)	222 (12.07)	192 (12.08)	163 (9.57)	0.003*
Cholesterol‐lowering agents	6186 (95.77)	1280 (96.39)	1774 (96.47)	1501 (94.46)	1631 (95.77)	0.017
Hypoglycemic agents	1647 (25.50)	479 (36.07)	473 (25.72)	364 (22.91)	331 (19.44)	<0.001*
rt‐PA intravenous thrombolytic	625 (9.64)	128 (9.59)	161 (8.71)	162 (10.18)	174 (10.18)	0.401
Mechanical thrombectomy	16 (0.25)	3 (0.22)	7 (0.38)	3 (0.19)	3 (0.18)	0.592
Complications, *n* (%)						
Pulmonary infection	399 (6.15)	111 (8.31)	108 (5.84)	82 (5.15)	98 (5.73)	0.002*
NIHSS score at discharge, median (IQR)	2 (0–4)	2 (0–4)	2 (0–4)	2 (0–3)	2 (0–4)	0.062

*Note*: Kruskal‐Wallis test was used for continuous variables and Chi‐square test or Fisher's exact test for categorical variables.

Abbreviations: BMI, body mass index; eGFR, estimated glomerular filtration rate; FPG, fasting plasma glucose; HDL, high‐density lipoprotein; IQR, interquartile range; LDL, low‐density lipoprotein; mRS, modified Rankin Scale; NIHSS, the National Institutes of Health Stroke Scale; rt‐PA, recombinant tissue plasminogen activator; TC, total cholesterol; TG, triglyceride; TIA, transient ischemic attack.

* *p* value for trend <0.05.

### 3.2 Association of magnesium with all‐cause mortality and poor functional outcome

At the 3‐month assessment, 1003 (15.47%) patients experienced poor functional outcome (mRS score 3–6) and 1850 (28.54%) with mRS score 2–6; the proportion of patients who died was 1.71% (111). At the 1‐year follow‐up, 969 (14.95%) patients experienced poor functional outcome (mRS score 3–6) and 1706 (26.31%) with mRS score 2–6; 3.59% (233) patients died.

**FIGURE 1:**
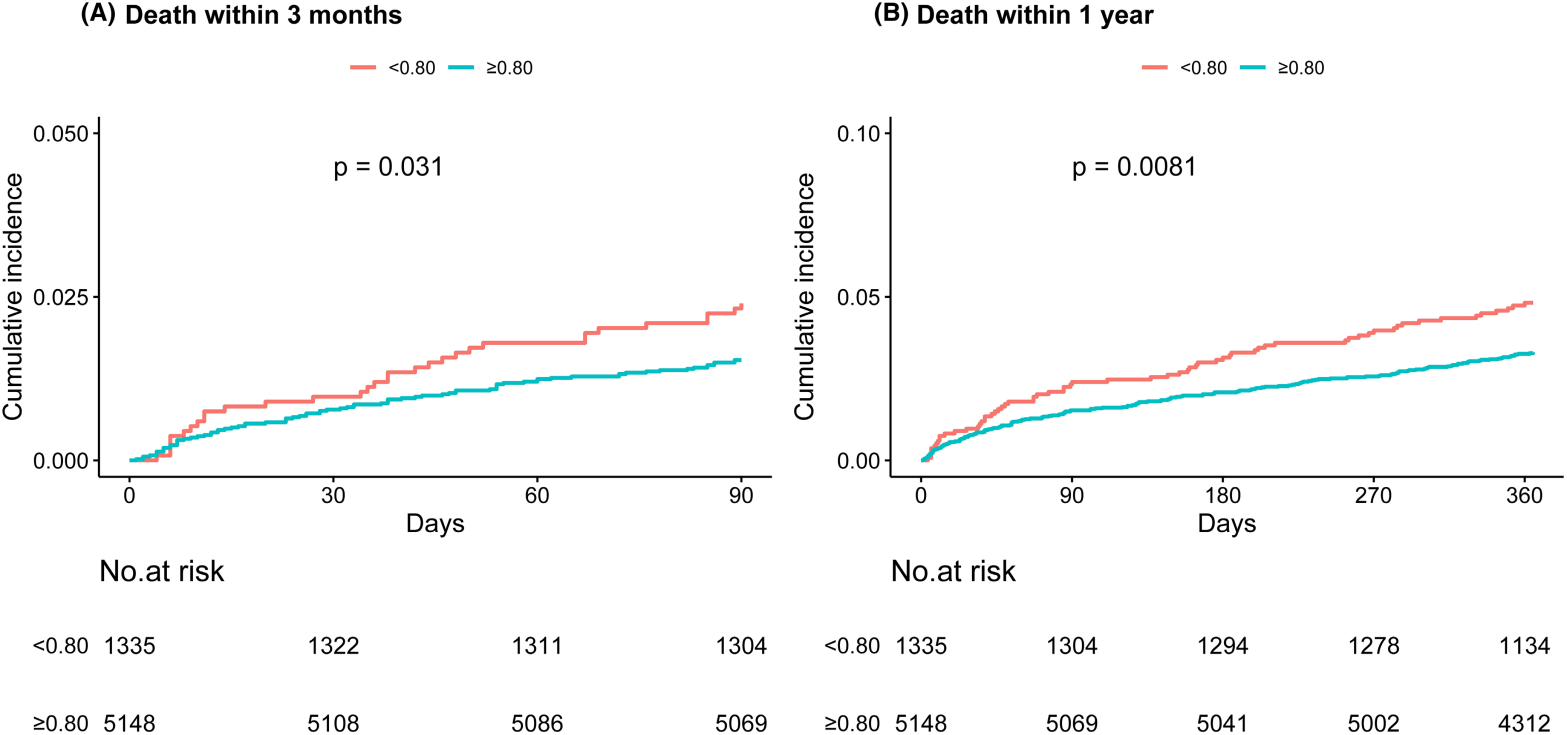
Kaplan‐Meier curves for clinical outcomes. (A) Kaplan‐Meier curve for all‐cause mortality within 3 months. (B) Kaplan‐Meier curve for all‐cause mortality within 1 year

The Kaplan‐Meier curves showed a significantly higher cumulative incidence of all‐cause mortality within 3 months and 1 year in patients with lower serum magnesium level (log‐rank *p* < 0.05, Figure 1). Table 2 shows the associations between serum magnesium levels and clinical outcomes. Compared with the fourth quartile group, the age‐ and sex‐adjusted HR of the first quartile of serum magnesium was 1.40 (95% CI: 1.02–1.93) for all‐cause mortality within 1 year, but when the model was further adjusted for all the potential variables, the association became insignificant (HR: 1.03; 95% CI: 0.71–1.50). Similar results were observed in the analysis of two groups of serum magnesium.

**TABLE 2:** Associations of serum magnesium with all‐cause mortality and poor functional outcomes

Outcomes	Quartiles of serum magnesium				*p* for trend	Binary classification	
	Q1	Q2	Q3	Q4		<0.8	≥0.80
At 3 months							
Death							
*n* (%)	32 (2.40)	32 (1.73)	21 (1.32)	26 (1.52)		32 (2.40)	79 (1.53)
Unadjusted	1.58 (0.91–2.73)	1.14 (0.65–1.99)	0.87 (0.50–1.51)	Reference	0.065	1.57 (0.95–2.58)	Reference
Model 1	1.61 (0.96–2.70)	1.18 (0.68–2.06)	0.91 (0.52–1.57)	Reference	0.046	1.55 (0.99–2.43)	Reference
Model 2	1.02 (0.61–1.72)	1.19 (0.68–2.07)	0.90 (0.54–1.51)	Reference	0.710	0.99 (0.66–1.48)	Reference
Model 3	0.99 (0.59–1.68)	1.10 (0.62–1.95)	0.80 (0.46–1.37)	Reference	0.759	1.02 (0.70–1.47)	Reference
mRS score 3–6							
*n* (%)	255 (19.10)	271 (14.66)	221 (13.89)	256 (14.98)		255 (19.10)	748 (14.53)
Unadjusted	1.34 (1.03–1.74)	0.98 (0.77–1.24)	0.92 (0.74–1.13)	Reference	0.039	1.39 (1.11–1.74)	Reference
Model 1	1.37 (1.07–1.76)	1.00 (0.78–1.27)	0.93 (0.75–1.16)	Reference	0.020	1.40 (1.14–1.73)	Reference
Model 2	1.27 (1.01–1.60)	0.96 (0.74–1.25)	0.95 (0.73–1.25)	Reference	0.086	1.31 (1.08–1.59)	Reference
Model 3	1.30 (1.02–1.64)	0.97 (0.75–1.25)	0.97 (0.74–1.28)	Reference	0.078	1.32 (1.09–1.61)	Reference
mRS score 2–6							
*n* (%)	450 (33.71)	533 (28.84)	403 (25.33)	464 (27.15)		450 (33.71)	1400 (27.20)
Unadjusted	1.36 (1.11–1.68)	1.09 (0.91–1.30)	0.91 (0.78–1.06)	Reference	0.003	1.36 (1.15–1.61)	Reference
Model 1	1.39 (1.14–1.71)	1.11 (0.93–1.33)	0.92 (0.79–1.08)	Reference	0.001	1.38 (1.17–1.62)	Reference
Model 2	1.29 (1.04–1.59)	1.09 (0.90–1.32)	0.93 (0.77–1.13)	Reference	0.014	1.27 (1.09–1.48)	Reference
Model 3	1.29 (1.04–1.59)	1.09 (0.90–1.31)	0.94 (0.78–1.14)	Reference	0.017	1.27 (1.09–1.48)	Reference
At 1 year							
Death							
*n* (%)	64 (4.79)	62 (3.35)	47 (2.95)	60 (3.51)		64 (4.79)	169 (3.28)
Unadjusted	1.37 (0.98–1.93)	0.96 (0.67–1.36)	0.84 (0.60–1.17)	Reference	0.071	1.47 (1.12–1.94)	Reference
Model 1	1.40 (1.02–1.93)	0.98 (0.69–1.40)	0.87 (0.62–1.20)	Reference	0.047	1.47 (1.14–1.89)	Reference
Model 2	1.06 (0.74–1.52)	0.96 (0.66–1.38)	0.86 (0.62–1.19)	Reference	0.668	1.13 (0.86–1.48)	Reference
Model 3	1.03 (0.71–1.50)	0.92 (0.63–1.35)	0.80 (0.57–1.13)	Reference	0.745	1.14 (0.87–1.49)	Reference
mRS score 3–6							
*n* (%)	233 (17.45)	281 (15.21)	210 (13.20)	245 (14.34)		233 (17.45)	736 (14.30)
Unadjusted	1.26 (0.96–1.66)	1.07 (0.86–1.34)	0.91 (0.76–1.09)	Reference	0.051	1.27 (0.99–1.62)	Reference
Model 1	1.30 (1.00–1.69)	1.11 (0.88–1.39)	0.93 (0.77–1.13)	Reference	0.024	1.28 (1.03–1.60)	Reference
Model 2	1.14 (0.88–1.47)	1.08 (0.85–1.36)	0.93 (0.76–1.15)	Reference	0.217	1.13 (0.91–1.40)	Reference
Model 3	1.13 (0.88–1.46)	1.07 (0.85–1.35)	0.94 (0.77–1.16)	Reference	0.241	1.12 (0.91–1.38)	Reference
mRS score 2–6							
*n* (%)	412 (30.86)	499 (27.00)	384 (24.14)	411 (24.05)		412 (30.86)	1294 (25.14)
Unadjusted	1.41 (1.14–1.75)	1.17 (0.97–1.41)	1.00 (0.85–1.18)	Reference	0.001	1.33 (1.11–1.60)	Reference
Model 1	1.46 (1.18–1.80)	1.20 (0.99–1.46)	1.03 (0.86–1.23)	Reference	<0.001	1.35 (1.14–1.60)	Reference
Model 2	1.34 (1.09–1.64)	1.18 (0.98–1.44)	1.05 (0.87–1.26)	Reference	0.005	1.24 (1.06–1.44)	Reference
Model 3	1.34 (1.10–1.64)	1.18 (0.98–1.42)	1.06 (0.88–1.27)	Reference	0.004	1.24 (1.06–1.44)	Reference

*Note*: ORs were used for mRS score 3–6 and mRS score 2–6. Model 1: adjusted for age and sex. Model 2: adjusted for age, sex, body mass index, current smoker, current alcohol drinking, disease history (diabetes, atrial fibrillation, coronary heart disease), the National Institutes of Health Stroke Scale score at admission, stroke etiology, anticoagulant agents, cholesterol‐lowering agents, hypoglycemic agents, and pulmonary infection. Model 3: adjusted for variables in model 2, plus total cholesterol, triglyceride, high‐density lipoprotein, low‐density lipoprotein, estimated glomerular filtration rate, fasting plasma glucose, and hospital stay.

Abbreviation: mRS, modified Rankin Scale.

**FIGURE 2:**
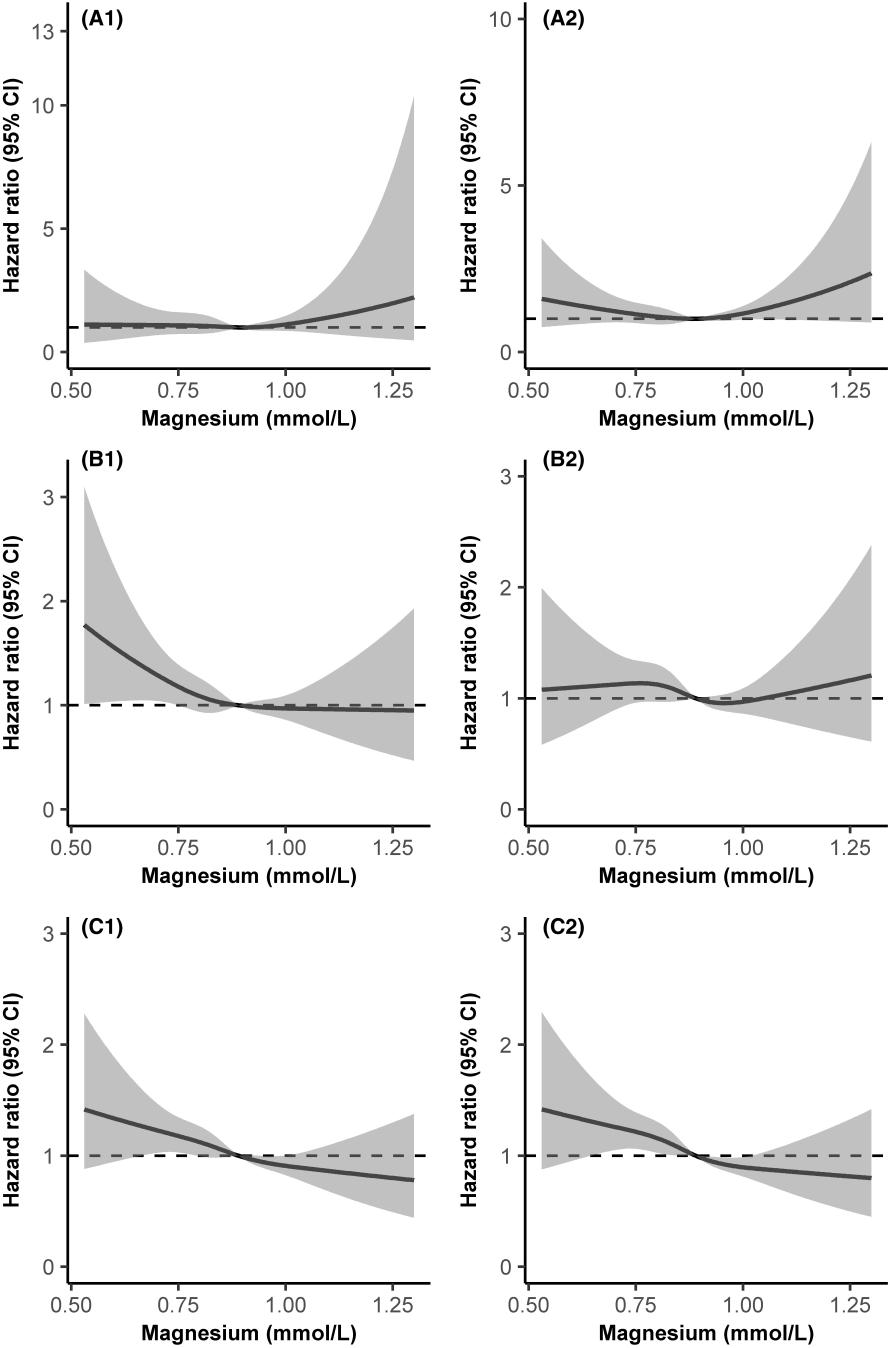
Association between serum magnesium and clinical outcomes. (A1) All‐cause mortality within 3 months. (A2) All‐cause mortality within 1 year. (B1) Poor functional outcome (mRS score 3–6) at 3 months. (B2) Poor functional outcome (mRS score 3–6) at 1 year. (C1) Poor functional outcome (mRS score 2–6) at 3 months. (C2) Poor functional outcome (mRS score 2–6) at 1 year. Adjusted for age, sex, body mass index, current smoker, current alcohol drinking, disease history (diabetes, atrial fibrillation, coronary heart disease), the National Institutes of Health Stroke Scale score at admission, stroke etiology, anticoagulant agents, cholesterol‐lowering agents, hypoglycemic agents, pulmonary infection, total cholesterol, triglyceride, high‐density lipoprotein, and low‐density lipoprotein, estimated glomerular filtration rate, fasting plasma glucose, and hospital stay.

**FIGURE 3:**
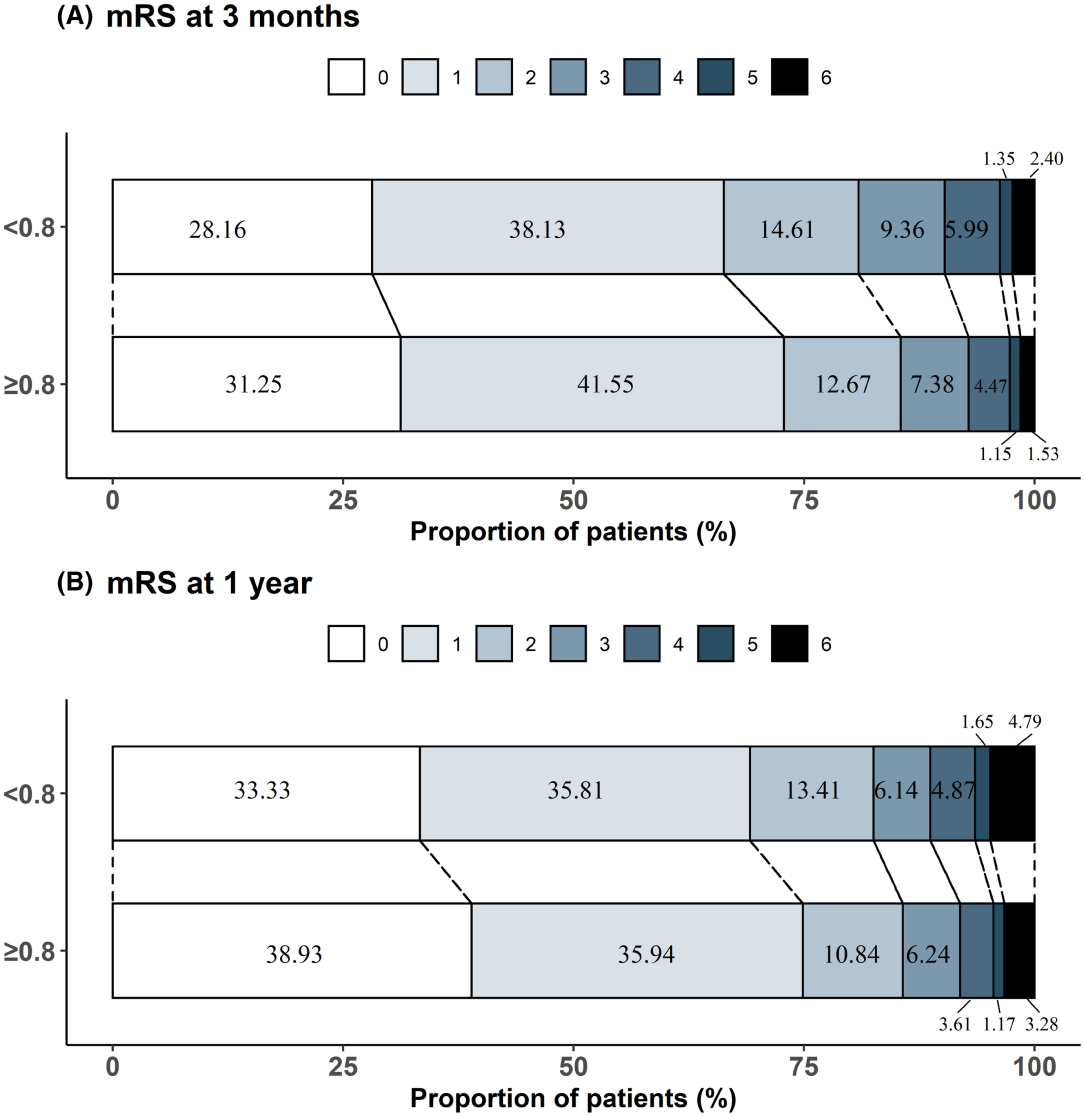
Distribution of modified Rankin scale score between serum magnesium groups at 3 months and 1 year

Multivariable‐adjusted spline regression models showed somewhat L‐shaped associations between serum magnesium and poor functional outcomes; as the serum magnesium level increased, the ORs of mRS score 3–6/2–6 decreased steadily at both 3 months and 1‐year follow‐up (Figure 2). After adjusting for the potential variables, patients in the first quartile of serum magnesium were significantly associated with increased risk of mRS score 3–6 and mRS score 2–6 at 3 months (OR: 1.30; 95% CI: 1.02–1.64; OR: 1.29; 95% CI: 1.04–1.59), when those in the fourth quartile group taken as reference. In the analysis of two groups of serum magnesium, patients with serum magnesium <0.80 mmol/L had 32% and 27% higher risk of poor functional outcome (mRS score 3–6/mRS score 2–6) at 3 months compared with patients with serum magnesium ≥0.80 mmol/L; the adjusted ORs were 1.32 (95% CI: 1.09–1.61) and 1.27 (95% CI: 1.09–1.48). Similar results were observed for mRS score 2–6 at 1 year. There were significant shifts in the distributions of the mRS scores according to the two groups of serum magnesium (Figure 3), and the common OR was 1.14 (95% CI: 1.02–1.28) and 1.18 (95% CI: 1.05–1.32) for mRS scores at 3 months and 1 year, respectively.

When restricting all analyses to patients free of diabetes mellitus and with normal kidney function (eGFR > 60 ml/min/1.73 m^2^) or additional adjusting for serum sodium, potassium, and calcium, all the above associations were not altered (Table 3).

**TABLE 3:** Sensitivity analysis of associations between serum magnesium and clinical outcomes

Outcomes	Quartiles of serum magnesium				*p* for trend	Binary classification	
	Q1	Q2	Q3	Q4		<0.8	≥0.80
At 3 months							
Death							
Sensitivity analysis 1	0.88 (0.53–1.48)	1.07 (0.61–1.88)	0.80 (0.47–1.36)	Reference	0.793	0.91 (0.63–1.32)	Reference
Sensitivity analysis 2	0.89 (0.45–1.77)	0.97 (0.47–2.00)	0.84 (0.44–1.60)	Reference	0.360	0.95 (0.56–1.60)	Reference
mRS score 3–6							
Sensitivity analysis 1	1.26 (0.99–1.61)	0.95 (0.73–1.23)	0.97 (0.74–1.28)	Reference	0.110	1.30 (1.06–1.59)	Reference
Sensitivity analysis 2	1.37 (1.03–1.82)	0.97 (0.72–1.32)	1.03 (0.72–1.48)	Reference	0.050	1.37 (1.06–1.77)	Reference
mRS score 2–6							
Sensitivity analysis 1	1.26 (1.02–1.57)	1.07 (0.89–1.30)	0.94 (0.78–1.13)	Reference	0.025	1.25 (1.07–1.46)	Reference
Sensitivity analysis 2	1.26 (0.99–1.59)	0.97 (0.80–1.17)	0.98 (0.79–1.21)	Reference	0.101	1.28 (1.06–1.54)	Reference
At 1 year							
Death							
Sensitivity analysis 1	0.97 (0.66–1.42)	0.90 (0.62–1.32)	0.81 (0.57–1.15)	Reference	0.745	1.07 (0.82–1.41)	Reference
Sensitivity analysis 2	1.20 (0.79–1.82)	0.91 (0.55–1.50)	0.80 (0.48–1.33)	Reference	0.213	1.32 (0.96–1.82)	Reference
mRS score 3–6							
Sensitivity analysis 1	1.09 (0.84–1.41)	1.05 (0.84–1.31)	0.94 (0.77–1.16)	Reference	0.361	1.09 (0.88–1.35)	Reference
Sensitivity analysis 2	1.19 (0.86–1.65)	1.06 (0.80–1.40)	1.05 (0.79–1.40)	Reference	0.240	1.15 (0.84–1.57)	Reference
mRS score 2–6							
Sensitivity analysis 1	1.19 (1.01–1.40)	1.10 (0.96–1.27)	1.03 (0.90–1.17)	Reference	0.007	1.00 (0.76–1.32)	Reference
Sensitivity analysis 2	1.35 (1.04–1.75)	1.09 (0.88–1.35)	1.09 (0.85–1.39)	Reference	0.028	1.27 (1.03–1.58)	Reference

*Note*: Sensitivity analysis 1: adjusted for variables in model 3 of Table 2, plus serum sodium, serum potassium, and serum calcium in all patients. Sensitivity analysis 2: in a subset of patients free of diabetes mellitus and with normal kidney function (defined as an eGFR > 60 ml/min/1.73 m^2^).

All the associations were consistent in nearly all the prespecified subgroups (*p* for interactions >0.05; Figure S1), except that a significant heterogeneity was observed across BMI for mRS score 2–6 at 1 year (*p* for interaction = 0.002), and across NIHSS score at discharge for mRS score 3–6 at 3 months (*p* for interaction = 0.043).

## 4 DISCUSSION

In this prospective national cohort of AIS and TIA, we found that low serum magnesium was associated with an increased risk of poor functional outcome at 3 months and 1 year in patients with AIS or TIA. The associations persisted when we excluded patients with diabetes mellitus and abnormal kidney function or additionally adjusted for serum sodium, potassium, and calcium in a sensitivity analysis. Our findings highlight the clinical relevance of evaluating serum magnesium levels in patients with AIS or TIA at admission.

Magnesium is a well‐known neuroprotective agent,^29^ and its low serum level is associated with an increased risk of ischemic stroke.^17^, ^18^ However, evidence of the association between serum magnesium and poor prognosis after stroke is limited and inconsistent. Siegler et al. conducted a retrospective study involving 313 AIS patients from one center in New Orleans and found that patients who had low magnesium at admission or a reduction in magnesium within 24 h of admission were not at a higher risk of neurologic deterioration or functional disability at discharge.^24^ Another study in America reported a null association between serum magnesium at admission and discharge functional outcome.^30^ The association between serum magnesium and in‐hospital mortality after stroke has been studied previously in a Chinese study, patients with low serum magnesium (<0.82 mmol/L) appeared to have a 2‐fold increase in the risk of in‐hospital mortality.^22^ Another study in China defined a composite outcome as death during hospitalization or neurological deficiency (NIHSS ≥ 10) at discharge and identified a significant lower risk when comparing the highest with the lowest quartile of magnesium.^23^ In the Turkish study, no significant correlations were found between neurological deficit levels and serum magnesium levels in ischemic and hemorrhagic stroke patients.^25^ However, interestingly, they found that the cerebrospinal fluid magnesium levels in ischemic stroke patients who died within 7 days were significantly lower than ischemic stroke patients who survived.^25^ A study from Romania which included 40 patients suggested a relationship between a low serum magnesium concentration 48 h after the onset of ischemic stroke and the intensity of the neurological deficit assessed by NIHSS score.^31^ Cognitive impairment after stroke is a common but neglected consequence compared to other neurological disorders such as sensory or motor impairment.^32^ Serum magnesium is positively correlated with cognitive function in both animal and human studies.^33^, ^34^, ^35^ One hospital‐based study enrolled 327 acute ischemic stroke patients and found a significant association between low serum magnesium concentrations and 1‐month poststroke cognitive impairment.^36^ Our study found that patients with low serum magnesium had a higher risk of short‐term and long‐term poor functional outcome. Any discrepancies between these studies may be partially due to the small sample size, and differences in demographic characteristics, primary outcomes, and follow‐up time. However, our results showed that the associations of low serum magnesium with all‐cause mortality within 3 months and 1 year were not statistically significant after adjusting for all the potential variables. One possible explanation is that the patients in our study were relatively mild, and the median NIHSS score at admission was only 3 (IQR, 1–6). Despite this, the patients included in the current study have more vascular risk factors that could affect stroke outcome, including age,^37^ high BMI,^38^ hypertension,^39^ diabetes,^40^ etc., indicating that the risk of poor prognosis is relatively high, and we adjusted multiple vascular risk factors. Our findings are almost consistent with previous studies and further confirmed the neuroprotective role of magnesium regarding the short‐term and long‐term prognosis of stroke.

Almost all magnesium in the adult human body is found in bones, tissues, and organs, with only 1% present in the blood.^41^ Although serum magnesium accounts for a small part of the total magnesium in the human body, it is still often used as a biomarker to assess magnesium metabolism status in clinical practice. There is a positive correlation between serum magnesium concentration and dietary magnesium intake.^42^, ^43^ Epidemiological studies including meta‐analysis have shown that dietary magnesium intake is associated with a reduction in the risk of stroke, cardiovascular death, and an improvement of the neurologic performance of discharged stroke patients.^6^, ^8^, ^44^, ^45^, ^46^, ^47^, ^48^ In our study, we observed a significant interaction between BMI and magnesium for mRS score 2–6 at 1 year. The effect of low serum magnesium on mRS score 2–6 at 1 year was more pronounced among patients with BMI < 24 kg/m^2^. Although there were no interactions between BMI and magnesium for other outcomes, we also observed a similar trend. These results suggested that nutritional status may play an important role in the effect of magnesium on the prognosis of stroke. Therefore, increased magnesium intake may reduce the mortality or disability risk of stroke patients. Previous clinical trials have explored the role of magnesium supplement in AIS recovery, but reported controversial results.^49^, ^50^, ^51^ Large clinical trials are needed to further confirm the effect of magnesium intake on the prognosis of stroke patients.

The exact pathophysiological mechanism between low magnesium level and poststroke dysfunction remains unclear. However, the potential role of magnesium as a neuroprotective agent in acute ischemic stroke has been fully demonstrated. We propose several hypotheses. First, serum magnesium may serve as a biomarker of some prestroke pathological states, which seriously affect the prognosis such as hypertension and hyperglycemia,^52^ severe renal insufficiency,^53^ they are known risk factors affecting stroke prognosis and mortality.^54^, ^55^, ^56^ Secondly, magnesium deficiency can lead to the activation of the inflammatory system,^57^ possibly through the activation of NF‐kB (including transcriptional programs that lead to the development of pro‐inflammatory phenotypes) leading to injury and dysfunction of vascular endothelium,^58^ which then result in the occurrence and development of atherosclerotic lesions,^59^ then further triggers vascular calcification and promotes lipid accumulation in vascular plaques.^60^ Third, magnesium deficiency is related to thrombosis, and platelet activation is the premise of thrombosis. Serum magnesium can promote the excretion of platelet inhibitor pgi230 and enhance the cleavage of hypervascular hemophilia factor (prethrombotic glycoprotein),^61^ thereby inhibiting platelet adhesion and aggregation. Previous studies have shown that low magnesium levels significantly increase platelet‐dependent thrombosis in patients with stable coronary heart disease.^62^ Fourth, low magnesium can increase oxidative stress and chronic inflammation,^63^ which plays an important role in brain aging and cognitive decline,^64^ thus leading to impaired survival status and functional recovery.

There were several limitations in this study. First, residual confounders may exist in the current observational study. Although we have adjusted many confounding factors and performed various sensitivity analyses, we were unable to adjust some potential confounders such as dietary magnesium intake status and intensive care unit duration. In addition, recent studies reported some novel prognostic biomarkers associated with stroke outcomes, such as the middle cerebral artery pulsatility index,^65^ serum occludin levels,^66^ direct bilirubin,^67^ and stress‐induced hyperglycemia.^68^ However, we could not adjust them due to the lack of data. Therefore, we cannot conclude the causal relationship between magnesium and outcomes. Second, we only have single serum magnesium measured at baseline and no follow‐up serum magnesium measurement. We, therefore, cannot evaluate the influence of dynamic changes in serum magnesium on poor outcomes of stroke, which could provide more valuable information to understand the mechanism of the associations. Third, serum magnesium was measured in sub‐centers, and equipment heterogeneity may lead to biased results, while this may have little impact, because the equipment in each center was under strict quality control in daily use and all laboratory tests were based on standard technical manuals of CNSR III. Finally, selection bias might occur due to the exclusion of patients lacking baseline serum magnesium and follow‐up information, further validation is needed to confirm the results.

## 5 CONCLUSIONS

In summary, the study showed that low serum magnesium was associated with a high risk of poor functional outcome in patients with AIS or TIA. More well‐designed intervention studies are needed to confirm whether magnesium supplementation could improve the outcomes after stroke.

## AUTHOR CONTRIBUTIONS

Q.X. and L.H conducted the literature review, interpreted the data, and drafted the initial manuscript. Q.X. performed the statistical analyses. A.W. and X.M. designed the study and were involved in data interpretation and manuscript preparation. H.L., X.T., Y.Z., Y.Z., and X.Z. contributed to the acquisition of data. L.C., P. S., and Y.W. interpreted data, reviewed, and revised the manuscript. All authors have read and approved the submitted manuscript.

## FUNDING INFORMATION

This work was supported by the National Natural Science Foundation of China (81870905, U20A20358, 82111530203), Chinese Academy of Medical Sciences Innovation Fund for Medical Sciences (2019‐I2M‐5‐029), National Key Research and Development Program of China (2022YFC3600600, 2022YFC3600603), Training Fund for Open Projects at Clinical Institutes and Departments of Capital Medical University (CCMU2022KYXZ009), the Capital's Funds for Health Improvement and Research (2020‐1‐2041), and Beijing Municipal Administration of Hospitals Incubating Program (PX2020021).

## CONFLICTS OF INTEREST

None.

## Supporting information


Appendix S1

Figure S1

## Data Availability

The data that support the findings of this study are available on request from the corresponding author. The data are not publicly available due to privacy or ethical restrictions.
