# Supplementary material for: Low serum magnesium is associated with poor functional outcome in acute ischemic stroke or transient ischemic attack patients
Source: CNS Neurosci Ther. 2022 Nov 22;29(3):842–54. doi: 10.1111/cns.14020 (PMC9928556; doi:10.1111/cns.14020)
Supplement: Supplementary file 1 — Appendix S1 [file CNS-29-842-s002.docx]

**Supplemental Materials for**

**Low Serum Magnesium are Associated with** **Poor Functional Outcome in Acute Ischemic Stroke or Transient Ischemic Attack Patients**

**Running title: magnesium and outcomes after stroke**

Qin Xu,^1,2*^ Lele Hu,^3*^ Lu Chen, ^4^ Hao Li,^1,2^ Xue Tian,^5,6^ Yingting Zuo,^5,6^ Yijun Zhang,^1,2^ Xiaoli Zhang,^1,2^ Ping Sun,^3^ Yongjun Wang^1,2,7,8^ Anxin Wang,^1,2#^ and Xia Meng,^1,2#^

^1^ Department of Neurology, Beijing Tiantan Hospital, Capital Medical University, Beijing, China;

^2^ China National Clinical Research Center for Neurological Diseases, Beijing Tiantan Hospital, Capital Medical University, Beijing, China;

^3^ The Second People's Hospital of Guiyang, Guizhou, China;

^4^ Department of Neurology, ZiBo Central Hospital, Zibo, China;

^5^ Department of Epidemiology and Health Statistics, School of Public Health, Capital Medical University, Beijing, China;

^6^ Beijing Municipal Key Laboratory of Clinical Epidemiology, Beijing, China;

^7^ Advanced Innovation Center for Human Brain Protection, Capital Medical University, Beijing, China;

^8^ Center for Excellence in Brain Science and Intelligence Technology, Chinese Academy of Sciences, Shanghai, China.

*Qin Xu and Lele Hu contributed equally.

**^#^Corresponding Authors:**

1. Anxin Wang, PhD, Department of Neurology, Beijing Tiantan Hospital, Capital Medical University. China National Clinical Research Center for Neurological Diseases, Beijing Tiantan Hospital, Capital Medical University. No. 119 South 4th Ring West Road, Fengtai District, Beijing 100070, China. Email: wanganxin@bjtth.org

2. Xia Meng, MD, PhD, Department of Neurology, Beijing Tiantan Hospital, Capital Medical University. China National Clinical Research Center for Neurological Diseases, Beijing Tiantan Hospital, Capital Medical University. No. 119 South 4th Ring West Road, Fengtai District, Beijing 100070, China. Email: mengxia@ncrcnd.org.cn

# Table S1. Baseline characteristics between included and excluded patients

| **Characteristics** | **Total** | **Excluded** | **Included** | ***P* value** |
| --- | --- | --- | --- | --- |
| No. of the patients | 15166 | 8683 | 6483 |  |
| Serum magnesium, median (IQR), mmol/L | 0.87 (0.8-0.93) | 0.86 (0.79-0.92) | 0.87 (0.8-0.93) | 0.064 |
| Age, median (IQR), y | 63 (54-70) | 62 (54-70) | 63 (55-71) | <0.001 |
| Women, n (%) | 4802 (31.66) | 2726 (31.39) | 2076 (32.02) | 0.411 |
| BMI, median (IQR), kg/m^2^ | 24.49 (22.6-26.56) | 24.49 (22.76-26.54) | 24.49 (22.49-26.57) | 0.085 |
| Current smoker, n (%) | 4752 (31.33) | 2747 (31.64) | 2005 (30.93) | 0.351 |
| Current alcohol drinking, n (%) | 6797 (44.82) | 3955 (45.55) | 2842 (43.84) | 0.036 |
| **Medical History, n (%)** |  |  |  |  |
| Hypertension | 9494 (62.60) | 5366 (61.80) | 4128 (63.67) | 0.018 |
| Stroke or TIA | 3675 (24.23) | 2174 (25.04) | 1501 (23.15) | 0.007 |
| Diabetes | 3510 (23.14) | 1994 (22.96) | 1516 (23.38) | 0.544 |
| Dyslipidemia | 1191 (7.85) | 747 (8.60) | 444 (6.85) | <0.001 |
| Atrial fibrillation | 1019 (6.72) | 515 (5.93) | 504 (7.77) | <0.001 |
| Coronary heart disease | 1608 (10.60) | 927 (10.68) | 681 (10.50) | 0.734 |
| Heart failure | 94 (0.62) | 49 (0.56) | 45 (0.69) | 0.314 |
| Peripheral vascular disease | 118 (0.78) | 80 (0.92) | 38 (0.59) | 0.020 |
| Renal insufficiency | 131 (0.86) | 81 (0.93) | 50 (0.77) | 0.287 |
| **Admission stroke data** |  |  |  |  |
| Time from onset to hospital admission, h | 12 (3-32) | 12 (3-33) | 11 (3-31) | 0.329 |
| hospital stay, days | 13 (10-16) | 14 (11-16) | 13 (10-15) | <0.001 |
| NIHSS at admission, median (IQR) | 3 (1-6) | 3 (1-6) | 3 (1-6) | 0.005 |
| Prestroke mRS score 2-5, n (%) | 1344 (8.86) | 794 (9.14) | 550 (8.48) | 0.157 |
| Stroke subtype, n (%) |  |  |  | 0.845 |
| Ischemic stroke | 14146 (93.27) | 8102 (93.31) | 6044 (93.23) |  |
| TIA | 1020 (6.73) | 581 (6.69) | 439 (6.77) |  |
| Stroke etiology, n (%) |  |  |  | <0.001 |
| Large-artery atherosclerosis | 3856 (25.43) | 2286 (26.33) | 1570 (24.22) |  |
| Cardioembolism | 917 (6.05) | 467 (5.38) | 450 (6.94) |  |
| Small-vessel occlusion | 3165 (20.87) | 1906 (21.95) | 1259 (19.42) |  |
| Other determined etiology | 182 (1.20) | 77 (0.89) | 105 (1.62) |  |
| Undetermined etiology | 7046 (46.46) | 3947 (45.46) | 3099 (47.80) |  |
| **Laboratory data, median (IQR)** |  |  |  |  |
| TC, mmol/L | 4.14 (3.43-4.90) | 4.06 (3.38-4.80) | 4.24 (3.52-5.05) | <0.001 |
| TG, mmol/L | 1.37 (1.02-1.91) | 1.37 (1.03-1.89) | 1.37 (1.02-1.93) | 0.368 |
| HDL, mmol/L | 1.08 (0.91-1.29) | 1.08 (0.91-1.29) | 1.09 (0.91-1.30) | 0.059 |
| LDL, mmol/L | 2.44 (1.84-3.11) | 2.39 (1.80-3.03) | 2.52 (1.91-3.22) | <0.001 |
| eGFR, mL/min/1.73 m^2^ | 95.48 (83.16-104.76) | 95.85 (84.2-105.34) | 94.94 (82.04-104.2) | <0.001 |
| FPG, mmol/L | 5.52 (4.9-6.89) | 5.48 (4.86-6.8) | 5.6 (4.94-7) | <0.001 |
| **Medications in hospital, n (%)** |  |  |  |  |
| Antihypertensive agents | 7000 (46.50) | 3857 (44.88) | 3143 (48.66) | <0.001 |
| Antiplatelet agents | 14613 (97.08) | 8340 (97.04) | 6273 (97.12) | 0.785 |
| Anticoagulant agents | 1546 (10.27) | 785 (9.13) | 761 (11.78) | <0.001 |
| Cholesterol-lowering agents | 14506 (96.37) | 8320 (96.81) | 6186 (95.77) | <0.001 |
| Hypoglycemic agents | 3792 (25.19) | 2145 (24.96) | 1647 (25.50) | 0.450 |
| rt-PA intravenous thrombolytic | 1303 (8.59) | 678 (7.81) | 625 (9.64) | <0.001 |
| Mechanical thrombectomy | 39 (0.26) | 23 (0.26) | 16 (0.25) | 0.828 |
| **Complications, n (%)** |  |  |  |  |
| Pulmonary infection | 824 (5.43) | 425 (4.89) | 399 (6.15) | <0.001 |
| NIHSS score at discharge, median (IQR) | 1 (0-3) | 1 (0-3) | 2 (0-4) | <0.001 |

Abbreviations: BMI, body mass index; eGFR, estimated glomerular filtration rate; FPG, fasting plasma glucose; HDL, high-density lipoprotein; IQR, interquartile range; LDL, low-density lipoprotein. mRS, modified Rankin Scale; NIHSS, the National Institutes of Health Stroke Scale; rt-PA, recombinant tissue plasminogen activator; TC, total cholesterol; TG, triglyceride; TIA, transient ischemic attack; rt-PA, recombinant tissue plasminogen activator.

# Figure S1. Subgroup analysis of association between serum magnesium and clinical outcomes.
