# Supplementary figures and images for: Low serum magnesium is associated with poor functional outcome in acute ischemic stroke or transient ischemic attack patients
Source: CNS Neurosci Ther. 2022 Nov 22;29(3):842–54. doi: 10.1111/cns.14020 (PMC9928556; doi:10.1111/cns.14020)

A1-Death within 3 months

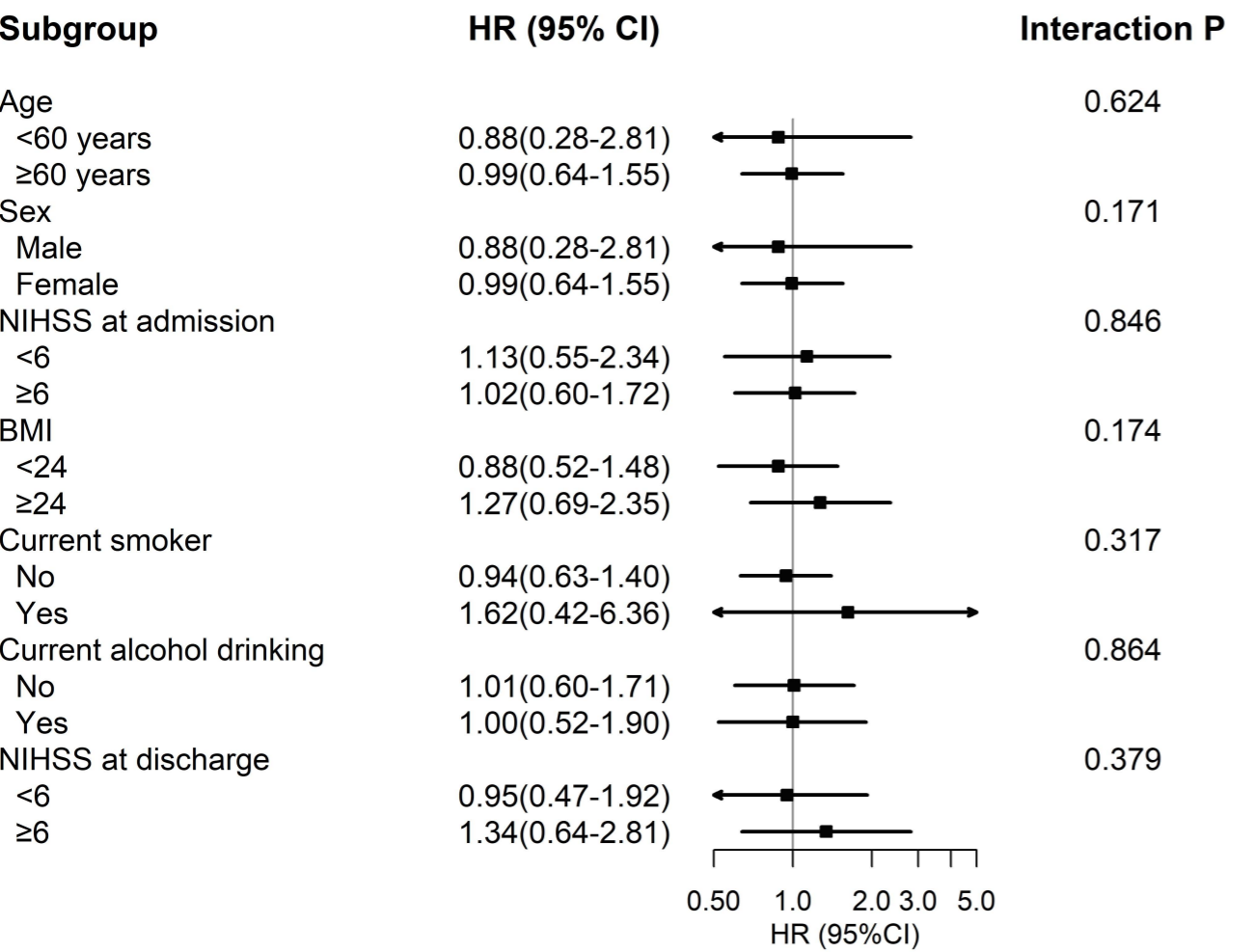

A2-Death within 1 year

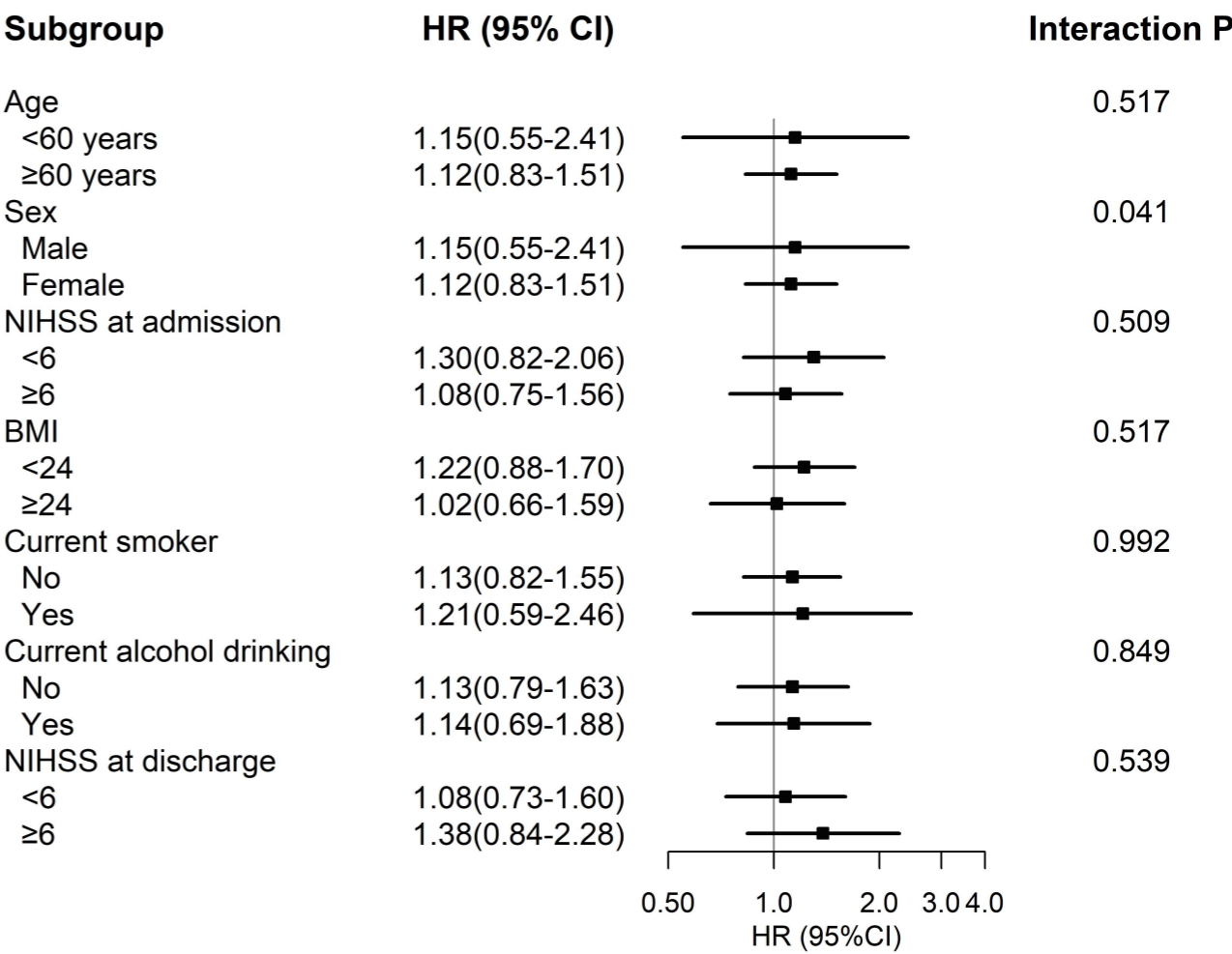

B1-mRS 3-6 at 3 months

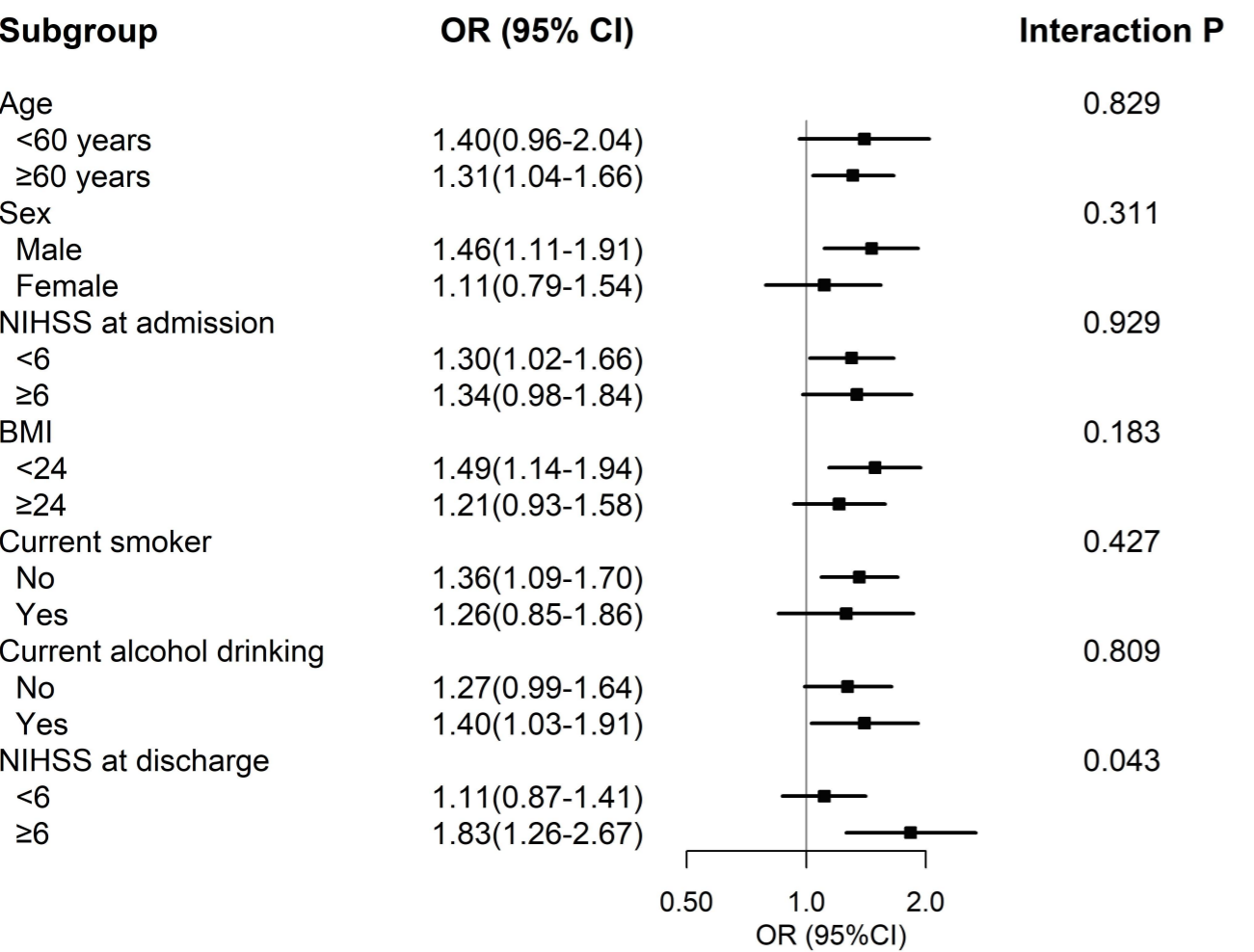

B2-mRS 3-6 at 1 year

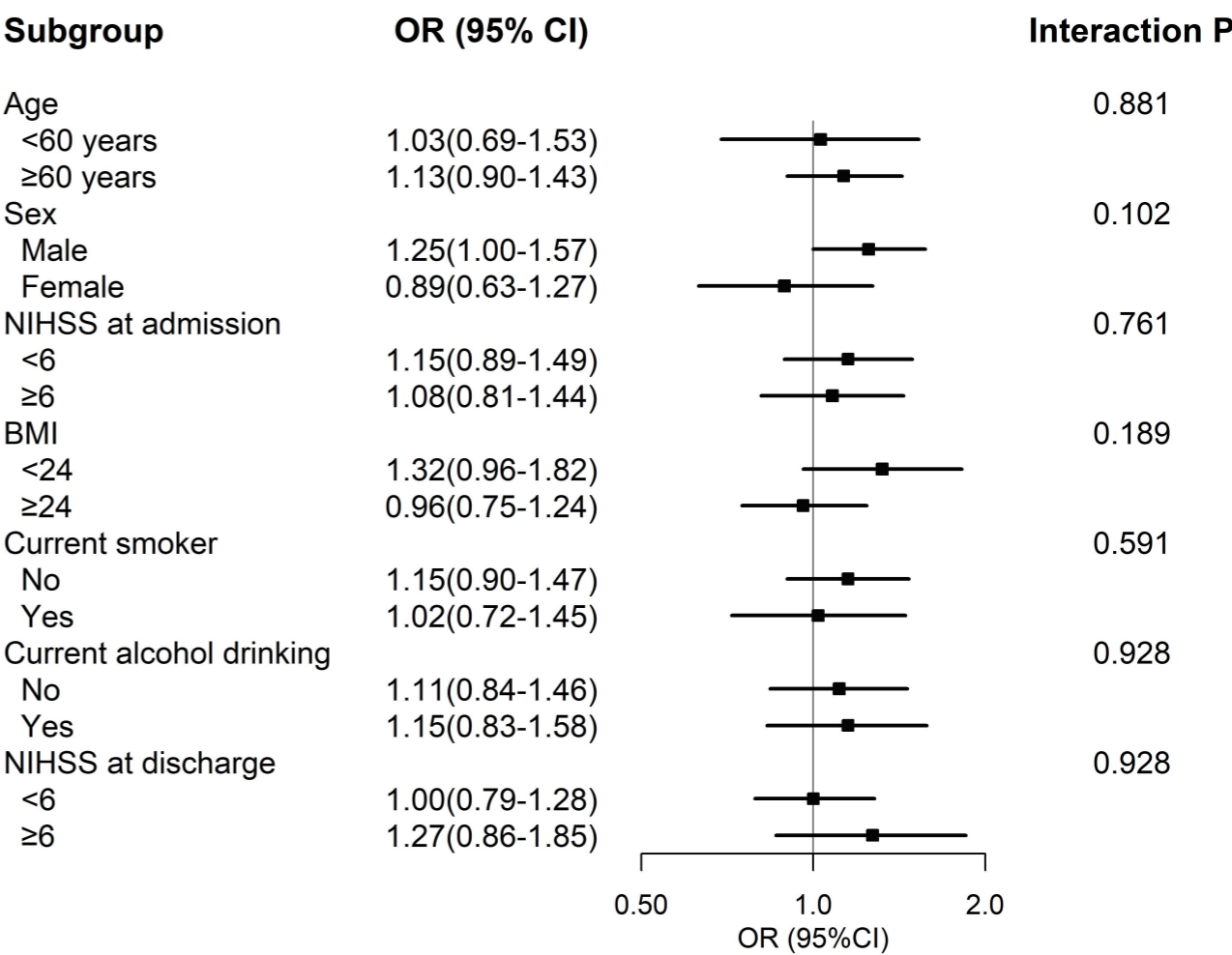

C1-mRS 2-6 at 3 months

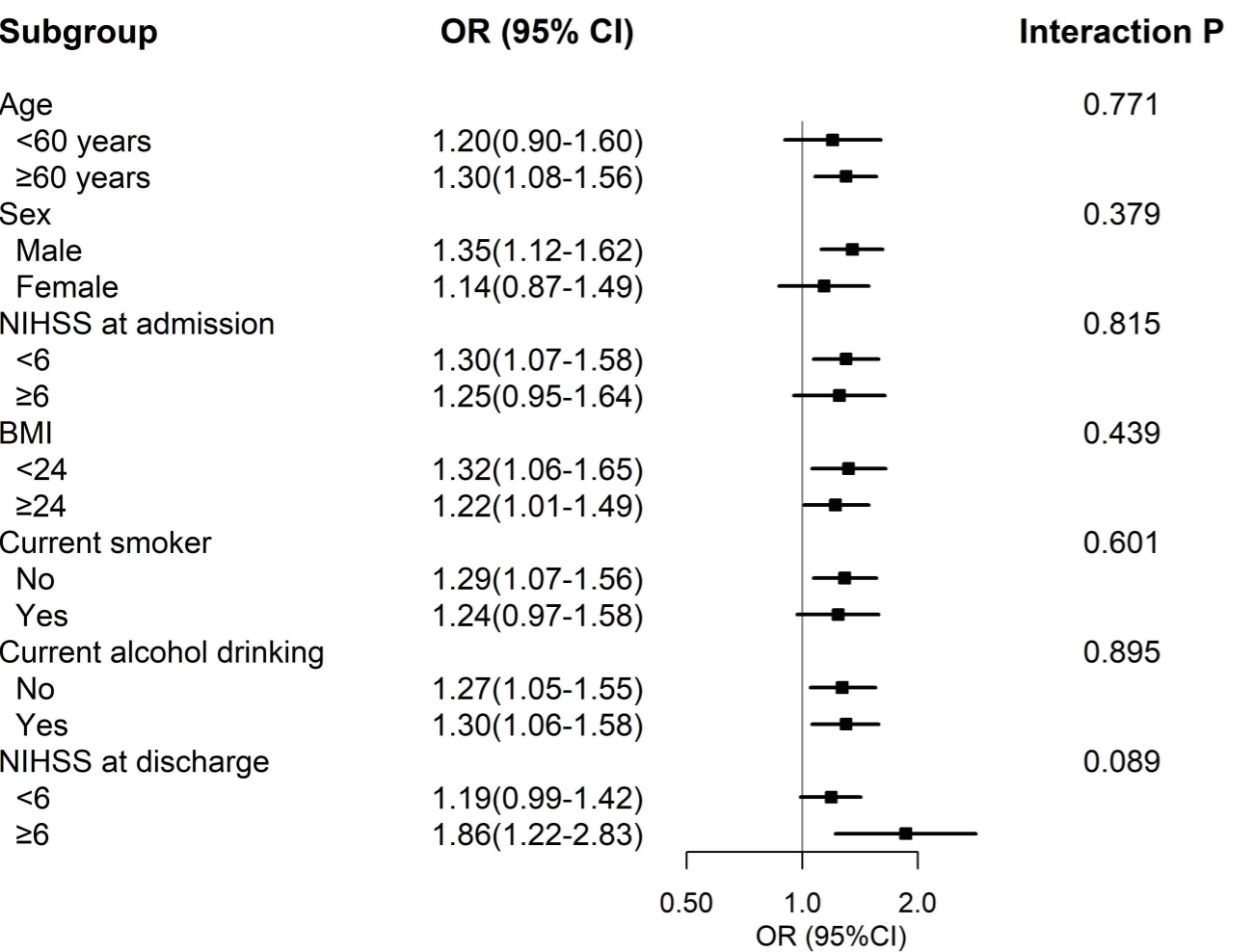

C2-mRS 2-6 at 1 year

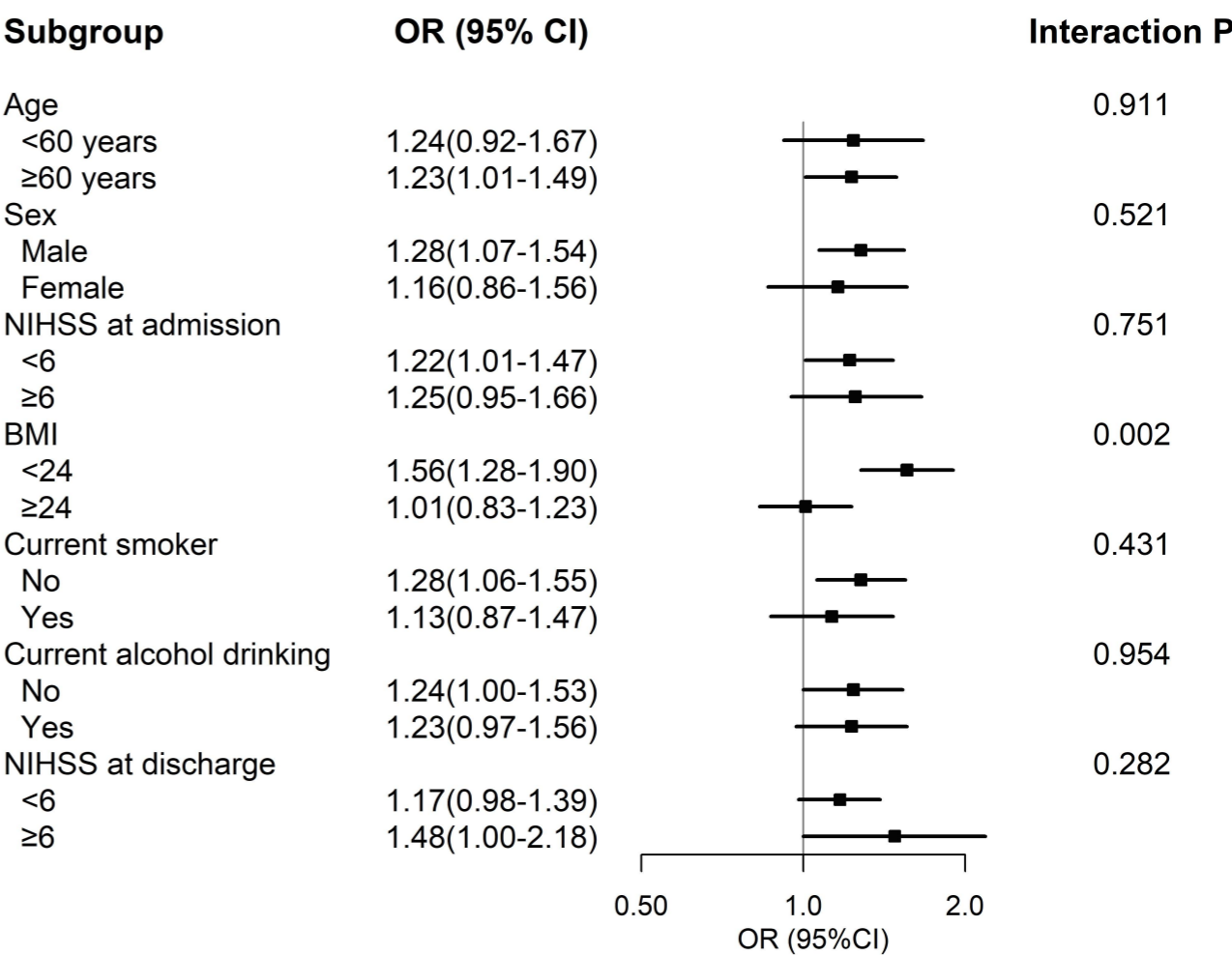

Supplement: Supplementary file 2 — Figure S1 [file CNS-29-842-s001.pdf]
